# Medication reconciliation in pediatrics: a validation of instruments to prevent medication errors

**DOI:** 10.1590/0034-7167-2021-0755

**Published:** 2023-03-06

**Authors:** Gabriela Almeida Aranha, Andréia Cascaes Cruz, Mavilde da Luz Gonçalves Pedreira

**Affiliations:** I Universidade Federal de São Paulo. São Paulo, São Paulo, Brazil

**Keywords:** Medication Reconciliation, Patient Safety, Pediatrics, Medication Errors, Professional-Family Relations., Conciliación de Medicamentos, Seguridad del Paciente, Pediatría, Errores de Medicación, Relaciones Profesional-Familia., Reconciliação de Medicamentos, Segurança do Paciente, Pediatria, Erros de Medicação, Relação Profissional-Família.

## Abstract

**Objectives:**

to develop and validate the content of two instruments for promoting medication reconciliation for the transition of care of hospitalized children.

**Methods:**

methodological study, conducted in five stages: scope review for conceptual structure; elaboration of the initial version; content validation with five specialists using the Delphi technique; reassessment; and construction of the final version of the instruments. A content validity index of at least 0.80 was adopted.

**Results:**

three rounds of evaluation were carried out to reach the validity index of the proposed contents, whereas a new analysis of 50% of the 20 items of the instrument aimed at families, and 28.5% of the 21 items aimed at professionals was necessary. The instrument aimed at families reached an index of 0.93, and the instrument for professionals, 0.90.

**Conclusions:**

the proposed instruments were validated. It is now possible to proceed with practical implementation studies to identify their influence on safety during medication reconciliation at transition of care.

## RESUMO

**Objetivos:**

elaborar e validar o conteúdo de dois instrumentos para a promoção da reconciliação medicamentosa na transição dos cuidados de crianças hospitalizadas.

**Métodos:**

estudo metodológico realizado em cinco etapas: revisão de escopo para estrutura conceitual; elaboração da versão inicial; validação do conteúdo com cinco especialistas utilizando a Técnica Delphi; reavaliação; e construção da versão final dos instrumentos. Adotou-se o índice de validade de conteúdo de, no mínimo, 0.80.

**Resultados:**

foram realizadas três rodadas de avaliação para alcance de índice de validade nos conteúdos propostos, sendo necessária nova análise de 50% dos 20 itens do instrumento destinado às famílias, e 28,5% dos 21 itens destinados aos profissionais. O instrumento direcionado às famílias atingiu índice de 0,93; e o instrumento, aos profissionais, 0,90.

**Conclusões:**

os instrumentos propostos foram validados, sendo possível prosseguir com estudo de implementação prática para identificar influência na segurança durante a reconciliação medicamentosa na transição dos cuidados.

## RESUMEN

**Objetivos:**

elaborar y validar contenido de dos instrumentos para la promoción de conciliación medicamentosa en la transición de atención de niños hospitalizados.

**Métodos:**

estudio metodológico realizado en cinco etapas: revisión de ámbito para estructura conceptual; elaboración de versión inicial; validación del contenido con cinco especialistas utilizando la Técnica Delphi; re-evaluación; y construcción de versión final de los instrumentos. Adoptado índice de validez de contenido, como mínimo, 0.80.

**Resultados:**

fueron realizadas tres rondas de evaluación para alcance de índice de validez en los contenidos propuestos, siendo necesario nuevo análisis de 50% de los 20 ítems del instrumento destinado a familias, y 28,5% de los 21 ítems destinados a profesionales. Instrumento dirigido a familias alcanzó índice de 0,93; y el instrumento, a profesionales, 0,90.

**Conclusiones:**

instrumentos propuestos fueron validados, siendo posible proseguir con estudio de implementación práctica para identificar influencia en la seguridad durante la conciliación medicamentosa en la transición de atención.

## INTRODUCTION

Patient safety is a goal to be achieved in any health care system, and medications errors is considered one of the most relevant risks within this context due to its high possibility of causing harm to the individual. As it is an avoidable event during drug therapy^(1-2)^, the multidisciplinary team should be aware of all the steps involved in this process to prevent medication errors. Errors of omission, dosage, dilution, frequency, or route of administration are described as the most frequent medication errors, and it is estimated that one in five patients is affected by some type of error^(3-4)^.

The pediatric population is considered one of the most prone to medication errors due to factors such as: medications not adapted for children, the need for fractioning and careful dosing calculation, and lack of communication with the family regarding home medication therapy. According to the World Health Organization (WHO)^(5)^, it is estimated that 50% of all medications are inappropriately prescribed, dispensed, or administered during and after pediatric hospitalization. Therefore, to prevent these types of errors, proper guidance and good patient-family-professional relationship are proposed as interventions, especially during the discharge planning of pediatric patients from health care services^(1,6)^.

One of the critical moments for the occurrence of medication errors is during the transition of care, which refers to the transfer of patients between sectors of the same service or to other levels of health care. An example of this is when the patient is transferred between services of a given hospital, from a tertiary care sector to the patient’s domicile, or during transfers to Primary and/or Secondary Health Care sectors^(7-8)^.

To prevent medication errors during the transition of care, the implementation of medication reconciliation is recommended. This strategy consists in obtaining a list of drugs, including those used by the patient at home and those recently prescribed, and updating it with each new prescription, so it can be as accurate as possible. The in-hospital and out-of-hospital sectors to which the patient is transferred should be informed about the drugs that have been kept, changed, or discontinued, to avoid errors of omission, dosage, frequency, interval, or route of administration, interaction, as well as drug incompatibility^(6,9)^. One of the necessary sources of information to carry out the medication reconciliation process is the patient and/or family. Specifically, by means of an interview conducted by a duly trained multiprofessional team, such as nurses, pharmacists, or physicians, they provide data for pharmacological and clinical analysis between the drugs used at home and those imminently prescribed. In the pediatric area, the presence of the family is indispensable. By providing opportunities for their active participation in the care process, one of the main factors that contribute to ensuring patient safety is established^(10)^.

The relationship between nurse and family is the core element of the Patient- and Family-Centered Care (PFCC) model. This model recognizes the whole family as the focus of care, ensuring a care based on four concepts, which are respect and dignity, information sharing, participation, and collaboration - and the application of these concepts in clinical practice is facilitated through continuous negotiation between the team’s professionals and the family^(11-12)^.

Considering some factors such as medication reconciliation as a strategy capable of mitigating medication errors, the participation of the family as a core element of this process, and the unavailability of instruments that enable the applicability of medication reconciliation, the need to develop instruments aimed at preventing medication errors during the transition of care of children from hospital to home was verified.

## OBJECTIVES

To develop and validate the content of two instruments for promoting medication reconciliation for the transition of care of hospitalized children.

## METHODS

### Ethical aspects

The study followed the precepts of resolution 466/2012 of the National Health Council (CNS) and was approved by UNIFESP’s Research Ethics Committee (REC). Research participants’ agreement was formalized by signing the Informed Consent Form (ICF).

### Study Type

Methodological research^(13)^ that followed the assumptions set by COSMIN (COnsensus-based Standards for the selection of health Measurement INstruments) for the development of health measurement instruments^(14)^.

### Methodological procedures

The research was developed in five stages: a scope review to establish the conceptual framework; elaboration of the initial version of the instruments; evaluation of the instruments by five experts; content validation; and construction of the final version of the instruments. The literature scoping review aimed to establish the conceptual framework, focusing on identifying the main medication reconciliation errors during transition of care, both in the hospital environment and at home, based on patient safety integrated with patient- and family-centered care. It was found that illegible prescriptions, abbreviations in prescriptions, language barriers, lack of communication skills, and multiple combinations of medications are some of the main factors leading to medication errors^(1)^.

Two instruments were proposed: one intended for families to be used during hospital-to-home care transition, and another intended for health professionals to be used during in-hospital care transitions. The instruments should be filled out by a physician, nurse, or pharmacist from the multidisciplinary team that cares for the child; in addition, a double check should be performed by another professional from one of the aforementioned professional categories. The instrument directed to the families was guided by principles of the Patient and Family Centered Care Model, so that participation, respect, information sharing, and collaboration were incorporated in the written communication. The instrument aims to provide the child’s family with a better understanding of the drug therapy discharge plan through a compilation of all information regarding the drug therapy.

To proceed with the first stage of the evaluation, the following was sent by e-mail to the specialists 23 days after the invitation was sent: 1) the IFC; 2) the full instruments; 3) a script that guided the evaluation of the instruments; 4) a document for the evaluation of the medication reconciliation instrument for families; and 5) a document for the evaluation of the medication reconciliation instrument for professionals. The second and third evaluation stages by the expert judges committee were performed 45 and 90 days, respectively, after the return of the first evaluation stage.

### Data source

A committee of expert judges was composed according to the following criteria: I) having a degree in the areas of nursing, pharmacy, or medicine; II) be a specialist (certification, degree, or professional practical experience of at least five years), in the areas of pediatrics, patient safety, or pharmacy; and III) currently acting or have acted in any of the stages of the pediatric/neonatal clinical practice medication process. For this study, it was decided to limit the sample to five specialists with proven mastery and experience in the subject, capable of providing an effective evaluation of the proposed instrument^(15)^. The judges were chosen by non-probabilistic convenience sampling based on the identification of academic or recognition of assistance in the area, as they were professionals who published, worked in assistance, and gave classes or lectures in meetings of specialists who discuss this theme. The experts were invited by e-mail in April 2020, and all of them agreed to participate in the research. The committee was then composed of a pediatrician, a pharmacist with a master’s degree and clinical experience, a nurse with a doctorate and clinical experience in pediatrics, a nurse with a master’s degree and experience in pediatrics, and a specialist nurse with a doctorate in neonatology. There is no consensus in the literature as to the number of judges, with a recommendation of 5 to 20 people participating in this process^(15)^.

### Data collection and organization

Once the first version of the instruments was elaborated, we went on to the process of validating the instrument’s content. The evaluation of the selected instruments covered the following dimensions: adequacy; objectivity; pertinence; language clarity; and understanding of the content. The Delphi technique was selected for the validation process, which consists of a systematized information judgment process using scaling methods^(16)^. This study adopted a Likert-type scale with a graduated score from 1 to 3, where: 1 = I disagree; 2 = I neither agree nor disagree; and 3 = I agree.

### Analysis of results

The analysis of results included the calculation of the content validity index (CVI), which is a method to assess the level of consensus among judges on a given item. The CVI was calculated for each item of the instrument and for the instrument in its entirety (global); to estimate the CVI of each item, the number of responses with a score of 3 (“I agree” in the Likert scale) was calculated, divided by the number of evaluators (five); the global CVI was calculated using the sum of the indexes obtained by each item, divided by the total number of items. A consensus of 80% was considered as valid, that is, a CVI of 0.80; in cases in which this value was not reached, other rounds of evaluation for the item were carried out with the specialists^(17)^.

## RESULTS

The medication reconciliation instruments for health professionals and families underwent three rounds of evaluation with the committee of judges. This is because the CVI analysis showed that some items required readjustment after the first and second rounds, as shown by the validity index expressed in Tables 1 and 2; and by the description of the judges’ suggestions in Table 3.

**Table 1:** List of items and content validity index in the validation of the medication reconciliation instrument aimed at families

Items	1^st^ round	2^nd^ round	3^rd^ round
1 Instrument title	1.00	1.00^*^	1.00^*^
2 Introductory message to the family	1.00	1.00^*^	1.00^*^
Identification data			
3 Child’s name	1.00	1.00^*^	1.00^*^
4 Age	1.00	1.00^*^	1.00^*^
5 Diagnosis(es)	0.60	0.60^**^	1.00^*^
6 Current date	1.00	1.00^*^	1.00^*^
7 Name of custodian	1.00	1.00^*^	1.00^*^
8 Comorbidities	-	-	1.00^**^
Child Allergy Information			
9 Drug Allergy	0.80	0.80^*^	0.80^*^
10 Food Allergy	0.80	0.80^*^	0.80^*^
11 Warning message to the family	1.00	1.00^*^	1.00^*^
Chart with information about the drugs			
12 Prescription	0.80	1.00^**^	1.00^*^
13 Administration	0.60	1.00^**^	1.00^*^
14 Method of preparation/administration	0.80	1.00^**^	1.00^*^
15 Chart Layout	0.40	1.00^**^	1.00^*^
Information on the use of teas and herbal medicines by the child			
16 Use of teas	0.60	0.80^**^	0.80^*^
17 Use of herbal medicines	0.60	0.80^**^	0.80^*^
18 Open field for describing questions, doubts, and considerations of the family	1.00	1.00^*^	1.00^*^
Identification of the professionals who carried out the reconciliation process			
19 First identification	0.60	0.80^**^	0.80^*^
20 Second identification	0.60	0.80^**^	0.80^*^
Global content validity index	0.80	0.91	0.93

** Item already reflecting judges’ suggestion;

* Item validated in the previous round.

**Table 2:** List of items and content validity index in the validation of the medication reconciliation instrument aimed at professionals

Items	1^st^ round	2^nd^ round	3^rd^ round
1 Instrument title	0.80	0.80^*^	0.80^*^
Identification data			
2 Child’s name	0.80	0.80^*^	0.80^*^
3 Date of Birth	1.00	1.00^*^	1.00^*^
4 Age	0.80	0.80^*^	0.80^*^
5 Weight	1.00	1.00^*^	1.00^*^
6 Height	0.80	0.80^*^	0.80^*^
7 Name of custodian	0.80	0.80^*^	0.80^*^
8 Diagnosis(es)	0.60	0.80^**^	1.00
9 Current date	1.00	1.00^*^	1.00^*^
10 Patient’s place of origin	0.80	1.00^**^	1.00^*^
11 Comorbidities	-	-	1.00^**^
12 Child Allergy Information	1.00	0.80^**^	0.80^*^
Chart with information about the prescription and medication reconciliation			
13 Prescription	1.00	1.00^*^	1.00^*^
14 Conducts	1.00	1.00^*^	1.00^*^
15 Drugs destined for home use	1.00	1.00^*^	1.00^*^
16 Chart Layout	0.80	0.80^*^	0.80^*^
Information on the use of teas and herbal medicines by the child			
17 Use of teas	0.80	0.80^*^	0.80^*^
18 Use of herbal medicines	0.80	0.80^*^	0.80^*^
19 Open field for observations	0.80	0.80	0.80^*^
Identification of the professionals who carried out the reconciliation process			
20 First identification	0.80	1.00^**^	1.00^*^
21 Second identification	0.80	1.00^**^	1.00^*^
Global content validity index	0.86	0.89	0.90

** Item already reflecting judges’ suggestion;

* Item validated in the previous round.

**Table 3:** Description of the judges’ suggestions and changes made in each item of the medication reconciliation instruments

Item	Judges’ comments/suggestions
Instrument aimed at Families	
5	Replace "Main Diagnosis" with “Diagnosis(es)".
8	Include the item “Comorbidities”.
12	Replace "frequency" with "How many times a day?”.
	Replace "fasting", "breakfast", "lunch”, and "dinner" meals with "morning", "afternoon”, and "evening" periods.
13	Replace the term "administration" with "at what time does he take it?"
14	Replace “Method of preparation" with “How to prepare it?".
15	Replace the chart’s background color.
16/17	Add “When was the last time he took it?”.
	Remove "usage recommendations".
19*/*20	Change the data presentation.
	Include the professional's "unit/sector".
Instrument aimed at Professionals	
8	Replace "Main Diagnosis" with “Diagnosis(es)".
10	Change to universal inpatient units.
	Add “others”.
11	Include the item “Comorbidities”.
12	Replace "drug allergy" with "allergies".
20/21	Change the data presentation.
	Include the professional's "unit/sector".

Next, Table 1 presents the content validity index of the instrument aimed at families in the three rounds of evaluation.

In the instrument aimed at families, 19 items were evaluated in the first round: eight items (about 42%) achieved a CVI of 1.00, and four items (21%) achieved a CVI of 0.80. Among the seven non-validated items, six (31.5%) obtained a CVI of 0.60, and one item (5.2%) was evaluated with a CVI of 0.40 (Table 1). The CVI of the instrument in the first round was 0.80. All experts’ suggestions were analyzed by the researchers, and the suggested changes aimed to cover the different perspectives of proposed changes. Even in items validated for its content, other considerations were raised by the specialists and, when considered pertinent and relevant, incorporated for a new stage of analysis.

The items reassessed in the second round received an overall CVI of 0.91. However, the item “Diagnosis(es)” showed the need for a new evaluation, since its CVI remained at 0.60; the suggestion for adequacy, which requested the inclusion of a complementary item for the indication of comorbidities or secondary diagnoses, was accepted, and consensus was obtained in the third round of evaluation.

Next, we present the validation of the medication reconciliation tool aimed at health professionals (Table 2).

According to Table 2, seven items (35%) were validated with a CVI of 1.00, 12 items (60%) with a CVI of 0.80, and one item (5%) did not reach the minimum index proposed; specifically, the item “Diagnosis”, where, as in the instrument for families, had items proposed to be added by the judges that covered other diagnoses and comorbidities of the child. The overall CVI of the instrument after the first assessment was 0.86. Non-validated items were reformulated by the researchers and, based on specialists’ suggestions and on scientific evidence, underwent changes and were resent for analysis in the next round. The items that, although having been validated, but still received suggestions that were considered relevant, were improved and submitted to a new validation stage. Among those was item 12 “Child Allergy Information”, which, exceptionally, had its CVI reduced from 1.00 to 0.80, due to the disagreement of one of the judges with the change. For the second round, these were the changes in the instrument aimed at professionals: the item “Diagnosis” was changed to “Diagnosis(es)”; the child’s place of origin was generalized, and “Other service” was included; a field was added for food allergies; and there was an adaptation in the arrangement of the information on the professionals involved.

Table 3 shows the changes in each item according to the judges’ suggestions and comments during the validation process.

Therefore, the medication reconciliation instrument aimed at families reached an overall CVI of 0.80 in the first round; 0.91 in the second round; and, after the third submission, 0.93. The instrument aimed at professionals achieved an overall CVI of 0.86 in the first submission; in the second, 0.89; and, after the third validation round, 0.90.

**Figure 1:**
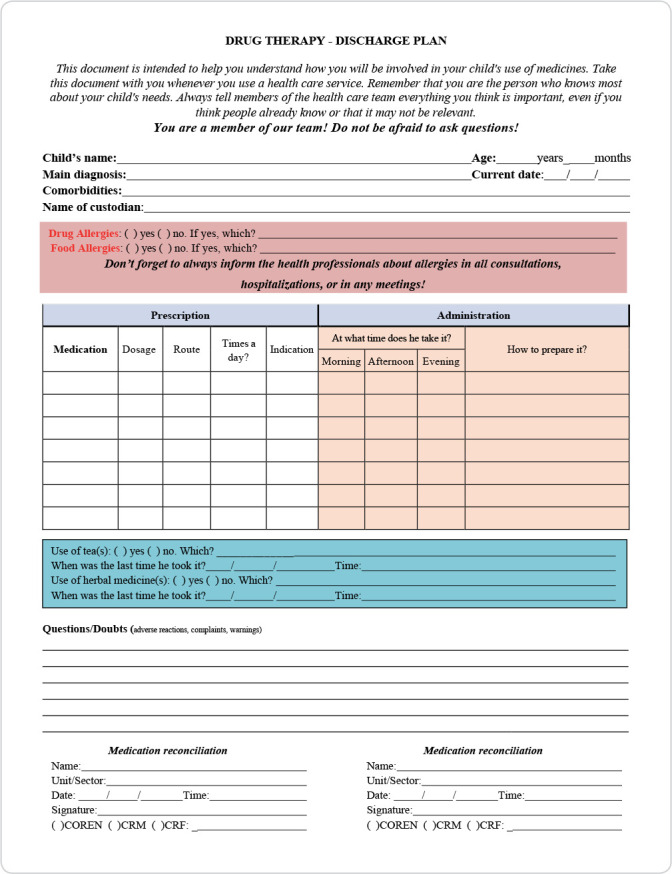
Instrument for medication reconciliation in pediatrics intended for use by family members

**Figure 2:**
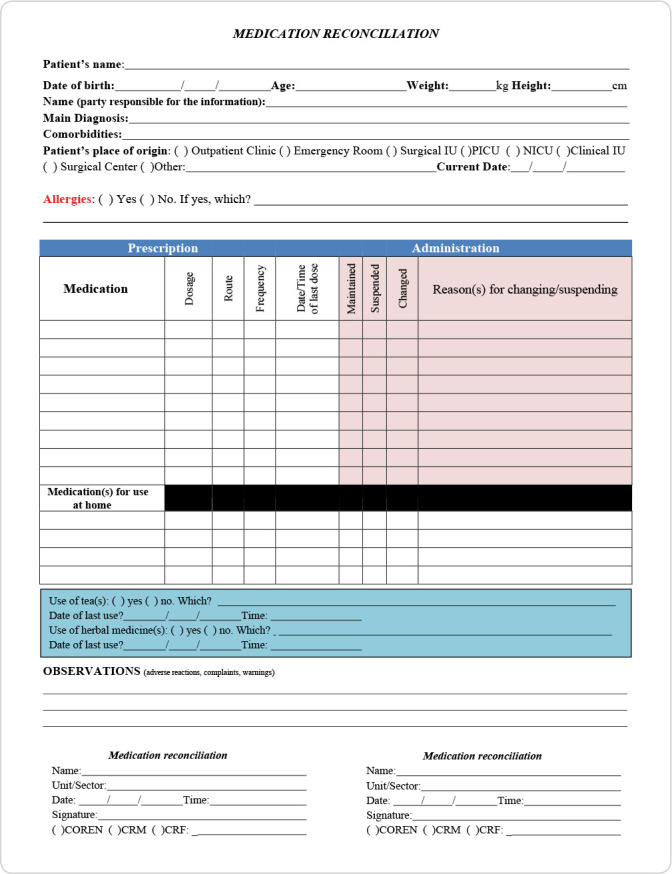
Instrument for medication reconciliation in pediatrics intended for use by health professionals - nurses, doctors, and pharmacists

Figures 1 and 2 show the instruments proposed and validated by the expert panel.

## DISCUSSION

To develop the proposed instruments, a search for evidence that demonstrated aspects related to the main medication errors in pediatrics and medication reconciliation was conducted. It was observed that the moments of greatest vulnerability for the occurrence of errors are related to the transition of care, particularly, the moments of hospital admission, intra-hospital transfer, and discharge; thus, ideal for applying the developed instruments^(18)^. After three rounds of validation with experts, it was possible to reach a consensus on the items that should be part of the instruments developed.

The proposed instruments were validated after three rounds of the Delphi technique, with an instrument CVI higher than 0.80 in all rounds. Revision was needed in items that were specifically related to the understanding of drug therapy constructs and in others related to child identification, with suggested changes in presentation. After this process, it was possible to provide instruments developed specifically for pediatric medication reconciliation performed by professionals and families, constituting an unprecedented proposition, but one that needs practical implementation to verify its effectiveness in reducing medication errors in pediatrics. During its use, the family should be informed that they should always carry this instrument with them during the child’s stay in health services, in Primary, Secondary, or Tertiary Care^(11)^. Thus, a strategy not yet found in other publications on the subject in the national literature^(19)^ is identified, one consistent with Anvisa’s National Plan for Patient Safety strategy, called “Patients for patient safety in health services”^(20)^.

In addition to the contents related to medication reconciliation to promote effectiveness of therapy and reduce the risk of medication errors identified in the literature review and refined by experts in the validation process, the instrument aimed at the family, in order to incorporate the principles of the PFCC, was formulated in an accessible language. The goal is for families to understand the information that will be provided and questioned, as well as feel encouraged to participate, question, and perceive themselves as essential members of the care team. To this end, phrases such as: “Always tell members of the health care team everything you think is important, even if you think people already know or that it may not be relevant. You are a member of our team! Don’t be afraid to ask!” The active participation of the patient and family in these moments is a way to reduce medication error as both have the right and room to speak before the treatment, and such participation makes them feel more at ease with the team, thus creating a relationship of respect and trust^(21)^.

In the instrument, there is a message with the purpose of alerting the family about the importance of communicating the team about the child’s allergies: “Don’t forget to always inform about drug allergies in all consultations, hospitalizations, or in any meeting with health professionals!” It is known that among adverse drug reactions, hypersensitivity is characterized by a reaction evidenced by non-predictable signs and symptoms after exposure to some drug. According to the study, the incidence of adverse reactions related to hypersensitivity in pediatric patients may go as high as 10%, with an increase in children under two years of age^(22)^.

Inadequate interprofessional communication is one of the main factors that influence the occurrence of medication errors^(23)^. The instruments developed are facilitators of professional-family communication, both verbal and written, so that the continuity of drug therapy happens. The gathering of important information in a single place enables a more appropriate and assertive management of drug therapy and allows a comprehensive assistance by allowing the professional to assess, in a single document, the updated information about the therapy^(24)^.

Currently, evidence on phytotherapeutic interventions is on the rise since the efficiency of the use of some medicinal plants and teas has already been scientifically proven. Therefore, if the treatment has a pharmacological action and, consequently, physiological effects, attention should be paid to the simultaneous use of other therapies: for example, chamomile leaf tea (*Matricaria chamomilla L./Chamomilla recutita L.*) interacts pharmacologically with Aspirin®, increasing the risk of bleeding in the patient. Thus, when considering the possibility of an adverse event related to drug therapy with possible harm to the patient, one should pay attention to the use of these substances during hospital admission and discharge^(25)^.

A recent study showed evidence on the use of an instrument capable of promoting drug reconciliation of patients in an oncology hospital at the time of admission. As a result, the instrument allowed the institution to identify the possible causes of medication errors and, consequently, their occurrence was reduced; the method also facilitated therapy follow-up for chronic diseases of the interviewed patients^(26)^. Similar results were observed in a study conducted in Australia, in which a medication reconciliation form was applied at the time of hospitalization, enabling the identification of recurrent discrepancies, with a consequent reduction in errors related to drug therapy^(27)^.

The WHO policy in the Global Action Plan for Patient Safety 2021 to 2030 stands out by envisioning the achievement of health care systems in which no patient is a victim of adverse events and in which all patients should receive safe and respectful care, at all times, and in all places of care. To achieve the greatest possible worldwide reduction in preventable adverse events resulting from unsafe health care, seven pillars of this policy stand out. Among them, the instruments presented here have the potential to contribute to the pillars of promoting safety in clinical processes, establishing policies to eliminate avoidable adverse events, and engaging patients and families as partners in care^(28)^.

The content validation performed allowed experts in the field to propose modifications within the developed instruments; and to suggest changes in the items that make up the medication reconciliation instruments by assessing the adequacy, objectivity, pertinence, clarity in language, and understanding of the content.

### Study limitations

A possible limitation of this study is the validation of the instrument by only five experts, since, despite their experience and mastery of the topic, it is necessary to expand the analysis to prove the relevance of the items and flows in achieving the objectives of the instrument.

Another limitation refers to the need to analyze the impact of the proposed instruments in the qualification of the medication reconciliation process in clinical practice: the influence of the procedural use of the instruments in preventing medication errors in pediatrics during the transition from in-hospital care to home care must be measured. Furthermore, the practical use by family members will allow the identification of pertinence and literacy level required for its use.

### Contributions to the field of Nursing

Medication reconciliation is an effective and efficient method to reduce medication errors and promote health safety. The proposed instruments can be widely used in the process of drug therapy in pediatrics at different levels of complexity and health care as they have low implementation costs and the potential to optimize drug therapy at times of transition of care.

It is assumed that these tools have the capacity to promote the sharing of information and encourage the active participation of patients and families in issues related to the use of medicines and the promotion of their own safety.

## CONCLUSIONS

The medication reconciliation instruments - one aimed at health professionals for use in transition of care in the in-hospital setting, and the other aimed at families for transition of care from hospital to home - were validated after an expert review process. It is possible to proceed with a practical implementation study to identify influence on safety during medication reconciliation in the transition of care, reducing medication errors through family participation.
